# Perceptions of pharmacists on the quality of automated blood pressure devices: a national survey

**DOI:** 10.1038/s41371-022-00670-4

**Published:** 2022-03-21

**Authors:** Dean S. Picone, Gregory M. Peterson, Shane L. Jackson, Norm R. C. Campbell, Christian Delles, Michael Hecht Olsen, Raj Padwal, Aletta E. Schutte, James E. Sharman

**Affiliations:** 1 Menzies Institute for Medical Research, University of Tasmania, Hobart, TAS Australia; 2 School of Pharmacy and Pharmacology, University of Tasmania, Hobart, TAS Australia; 3 Department of Medicine, Physiology and Pharmacology and Community Health Sciences, O’Brien Institute for Public Health and Libin Cardiovascular Institute of Alberta, University of Calgary, Calgary, AB Canada; 4 Institute of Cardiovascular and Medical Sciences, University of Glasgow, Glasgow, UK; 5 Department of Internal Medicine, Holbaek Hospital, Holbaek, Denmark; 6 Centre for Individualized Medicine in Arterial Diseases, Department of Regional Health Research, University of Southern Denmark, Odense, Denmark; 7 Department of Medicine, University of Alberta, Edmonton, AB Canada; 8 School of Population Health, University of New South Wales, Sydney, NSW Australia; 9 The George Institute for Global Health, Sydney, NSW Australia; 10 Hypertension in Africa Research Team (HART), Potchefstroom, South Africa; 11 Medical Research Council Unit for Hypertension and Cardiovascular Disease, North-West University, Potchefstroom, South Africa

**Keywords:** Risk factors, Hypertension

## Abstract

A recent study found that only 23.8% of blood pressure (BP) devices available for purchase from Australian pharmacies were validated for accuracy. The extent to which pharmacists are aware of this, and other issues related to the accuracy of BP devices, is not known and gathering this information was the aim of this study. An online survey of Australian pharmacists was distributed via the Pharmaceutical Society of Australia between 1 October and 25 November 2020. Questions were focused on the views of pharmacists related to the accuracy of BP devices. Two hundred and ten pharmacists completed the survey. The accuracy of BP devices sold by pharmacists was considered ‘quite’ or ‘extremely important’ to most respondents (94%). However, most respondents (90%) were unaware that less than one-quarter of BP devices sold by Australian pharmacies were validated, and this was ‘quite’ or ‘extremely surprising’ to many (69%). Many respondents (64%) associated a particular brand of BP device with greater accuracy. There was low awareness on proper ways to identify accurate BP devices, such as checking reputable online databases (43%). BP devices were stocked in respondents’ pharmacies based on perceived quality (50%), accuracy (40%), or as determined by the pharmacy chain (36%). In conclusion, providing accurate BP devices to consumers is important to pharmacists, but they were generally unaware that most devices available from pharmacies were not validated for accuracy. Pharmacist education, alongside advocacy for policies including regulations and strategic action, is required to ensure only validated BP devices are sold in Australia.

## Introduction

High blood pressure (BP) is the leading modifiable risk factor for cardiovascular disease and is responsible for over 10 million deaths globally each year [1]. Most early studies that provided evidence on the importance of high BP were performed using auscultatory BP measurements [2]. However, the accuracy of auscultatory BP is prone to error from incorrect user- and measurement processes [3]. To eliminate some of these errors and achieve more standardised BP measurements, automated BP devices are now recommended [4, 5].

Automated BP devices seek to derive BP values that are the same as auscultatory BP [6]. The accuracy of automated BP devices must be compared to auscultatory BP in accordance with a rigorous, internationally accepted scientific protocol [7, 8]. An automated BP device that fulfils the accuracy requirements set out in an accepted protocol is regarded as clinically validated. Validation testing is critically important because automated devices that are formally validated are more likely to have a higher degree of accuracy compared with those which are not validated or for which there is no evidence of validation [9–11].

In most countries, and also in Australia, evidence of proper clinical validation is not mandatory for an automated BP device to receive regulatory clearance (by the Therapeutic Goods Administration (TGA)) and be marketed for sale. Consequently, automated BP devices with unacceptable accuracy may be used for measurement of BP and subsequent clinical decisions, threatening appropriate management of hypertension. Using such devices could hinder attempts to reduce the risk of cardiovascular disease if the true BP is underestimated, whereas if the true BP is overestimated, patients could be overtreated and exposed to otherwise avoidable side effects of medication.

Recent work from Australia found that only 23.8% of automated BP devices sold online by pharmacies were validated [12]. This is a potential problem because pharmacists are trusted health professionals and a primary provider of BP devices to Australian consumers [13], but may be providing devices that have not been proven to be accurate. The extent to which pharmacists are aware of this is unknown and was one of the aims of this study. Furthermore, even though pharmacies tend to provide BP devices from well-known brands, accuracy cannot be assured or associated with a specific brand [14]. The extent to which pharmacists are aware, or undertake the process of checking validation status, as well as other issues related to the accuracy of BP devices and provision of advice to consumers, is not known. Thus, this study also aimed to better understand these issues via surveying the views of practicing Australian pharmacists.

## Methods

### Study overview

The online survey was conducted between 1 October 2020 and 25 November 2020 via the Research Electronic Data Capture portal [15]. The survey was distributed to members of the Pharmaceutical Society of Australia (PSA) via its emailed newsletter on 1 October 2020, with two reminders in subsequent newsletters, each spaced by a fortnight. There were no other selection criteria for the respondents.

The PSA is the only Australian Government-recognised national pharmacy organisation representing pharmacists in Australia. Survey responses were accepted until the day of online publication of an article containing information about the prevalence of validated BP devices in the *Australian Pharmacist* journal [16], which is the official journal of the PSA. It was not possible to determine the exact number of PSA members that the survey was distributed to (i.e. ‘approached for the research’). The study was approved by the Tasmanian Health and Medical Human Research Ethics Committee (reference H0023506). By submitting a completed survey, respondents provided consent for use of their responses in the research study.

This work has been conducted under the auspices of the Lancet Commission on Hypertension Group, who have recently published a position statement and recommendations with the aim of achieving greater global availability of validated BP devices [7]. The Group is also performing research studies to better understand factors contributing to the high prevalence of non-validated BP devices [12], and to identify potential solutions to this problem [17].

### Survey questions

The survey was developed by the investigator team and was then tested and revised based on the feedback of colleagues from the University of Tasmania, including those with qualitative research experience. The survey included participant demographic questions, including age range, sex, pharmacy qualifications, professional practice and geographic location in Australia. The survey also included questions related to attitudes and knowledge of BP devices and processes to determine device quality. Questions regarding measurement of BP in community pharmacies were also asked, with the purpose of understanding the methods and types of devices used. The survey questions were designed as either five-point Likert scale, true/false, multiple choice ‘check all that apply’ or open response fields. All true/false questions had ‘unsure’ and ‘other’ options (details in Supplementary Methods).

### Statistical analysis

Categorical data were reported as number of responses (percentage of total responses). Chi-square tests were used to examine whether pharmacist demographics were associated with certain responses to the BP specific questions. Chi-square analyses of the age range variable were performed by stratifying to age <50 years or ≥50 years. Chi-square tests of true/false questions about BP devices passing accuracy testing and TGA approval were analysed by combining the response options ‘false’, ‘unsure’ and ‘other’ into a single category. All statistical analyses were conducted using R version 3.5.1.

## Results

### Pharmacist demographics

Table 1 details the characteristics of respondents, who came from all states and territories of Australia. The age range of respondents was broad; 70% were female, and the sample appeared to be nationally representative in terms of age and sex (Table 1 and Supplementary Results) [18]. Respondents’ highest qualification was most commonly B. Pharm and most of their working time was spent in community pharmacy.

**Table 1:** Characteristics of the 210 pharmacists who completed the survey, as well as available national statistics [18].

Characteristics	*n* (%)	Australian statistics on pharmacists, *n* (%) (18)
Age		
<30 years	25 (12)	6052 (18)
30–39 years	54 (26)	12,789 (39)
40–49 years	46 (22)	6330 (19)
50–59 years	40 (19)	3844 (12)
60–69 years	31 (15)	2635 (8)
70+ years	12 (6)	1209 (4)
Prefer not to say	2 (1)	
Female sex	146 (70)	21,685 (63)
State/territory		
ACT	2 (1)	627 (2)
Queensland	28 (13)	6456 (20)
Tasmania	9 (4)	794 (3)
Victoria	57 (27)	8148 (26)
New South Wales	63 (30)	9610 (30)
South Australia	22 (11)	2241 (7)
Western Australia	27 (13)	3423 (11)
Northern Territory	1 (0.005)	256 (1)
Highest pharmacy qualification		
Bachelor of Pharmacy (standard degree)	144 (69)	
Honours (Hons)	19 (9)	
Ph.D.	4 (2)	
Masters	19 (9)	
Ph.C.	3 (1)	
Graduate Diploma	15 (7)	
Other	6 (3)	
Pharmacist registration year		
Prior to 1985	59 (28)	
1986 to 2000	46 (22)	
2001 to 2015	77 (37)	
2016 or later	27 (13)	

*HMRs* home medicines reviews, *RMMRs* residential medication management reviews.

### Attitudes toward BP monitors

**Fig. 1:**
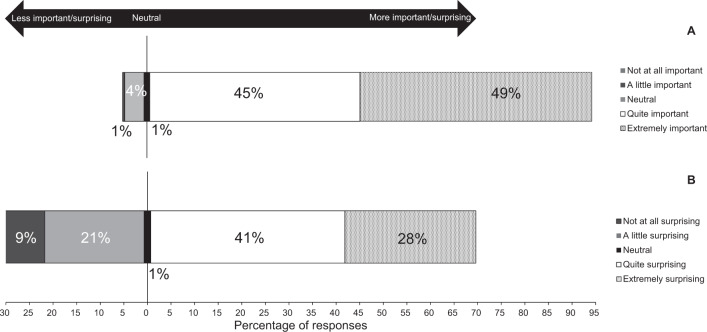
Responses to survey questions on knowledge of blood pressure device validation in Australia. Distribution of responses to the questions “Is the accuracy of blood pressure devices sold by pharmacists an issue of importance to you?” (**A**) and “Is this statistic (that 23.8% of blood pressure devices sold by pharmacies in Australia have passed accuracy testing according to rigorous scientific standards) surprising to you?” (**B**). Each respondent could only select a single option from five-point Likert scales.

Most respondents reported that the accuracy of BP devices sold by pharmacists was ‘quite’ (*n* = 94, 45%) or ‘extremely important’ (*n* = 103, 49%) to them (Fig. 1A). About half of the respondents believed all devices they sold were equally reliable in terms of measurement accuracy (*n* = 106, 51%). One-hundred and thirteen (54%) respondents mistakenly believed that BP devices must pass accuracy testing according to rigorous scientific standards before being sold in Australia, and 86 (41%) were unsure. One hundred and twenty-eight (62%) respondents mistakenly believed that a BP device approved by the TGA provided confirmation it had passed accuracy testing, while 64 (31%) respondents were unsure. Respondents were mostly unaware that only 23.8% of BP devices sold by pharmacies in Australia were validated (*n* = 188, 90%), and found this ‘quite’ or ‘extremely’ surprising (*n* = 144, 69%; Fig. 1B). Respondents younger than 50 years of age were more likely to be surprised than those aged 50 years or older (*χ*^2^ = 5.2, *p* = 0.023). Younger respondents were also more likely to mistakenly answer that BP devices approved for sale in Australia must pass accuracy testing (*χ*^2^ = 17.0, *p* < 0.0001) and that TGA approval of BP devices was confirmation that those devices had passed accuracy testing (*χ*^2^ = 6.4, *p* = 0.012).

### Awareness on identifying accurate BP monitors

**Fig. 2:**
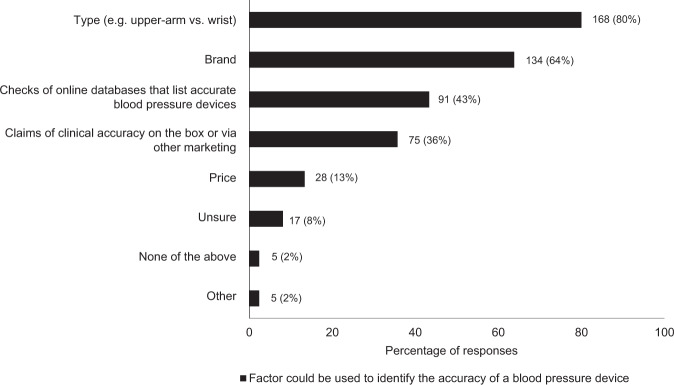
Prevalence of survey responses to factors that could be used to identify the accuracy of a blood pressure device. There was no limit on the number of factors a respondent could select. Data adjacent to each bar represent *n* (%).

**Fig. 3:**
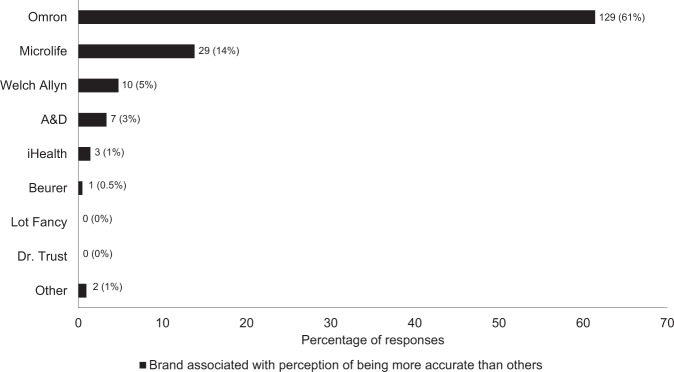
Brands of blood pressure devices and percentage of respondents who associated one brand with being more accurate than others (black segment of bars). There was no limit on the number of brands of devices a respondent could select. Data adjacent to each bar represent *n* (%).

There was low awareness of proper ways to determine the accuracy of BP devices, with only 91 (43%) respondents answering that checks of online databases that list accurate BP devices was an appropriate method. Respondents younger than 50 years of age more commonly indicated that claims of clinical accuracy on the device box or via other marketing could be used to identify the accuracy of a BP device (*χ*^2^ = 6.2, *p* = 0.013). On the other hand, pharmacy owners or managers were more likely than other respondents to correctly identify that checks of online databases listing accurate BP devices could be used to identify the accuracy of a BP device. Most respondents (*n* = 168, 80%) correctly associated greater accuracy with upper arm cuff, not wrist cuff, devices. Many respondents (*n* = 133, 64%) associated accuracy with a particular brand of device (Fig. 2). From a list of brands, one was most frequently selected by respondents as being associated with greater accuracy than other brands (*n* = 129, 61%; Fig. 3).

### Decisions about BP devices stocked in pharmacies

Respondents selected ‘quality’ of BP devices as the main reason that specific devices were stocked in their pharmacy (*n* = 104, 50%), indicating that perceived quality may drive the selection of devices to sell. 75 (36%) respondents had no control over the BP devices stocked in their pharmacies because this was determined by the pharmacy chain.

### Advice to patients buying BP devices

Most respondents stated that they provided advice on measuring BP at home to people buying BP monitors, either always (*n* = 155, 75%) or sometimes (*n* = 49, 24%). However, there was variability among respondents regarding the sources of advice provided, which included: general knowledge on BP (*n* = 146, 70%), information from the Heart Foundation (*n* = 118, 56%), and the Australian home BP consensus document [19] (*n* = 30, 14%). Other sources of home BP advice included in-house materials, BP device manufacturer advice, Royal Australian College of General Practitioners guidelines and the PSA Self Care Programme (*n* = 1 for each).

Respondents recommended that people purchasing BP devices at their pharmacies take their BP devices to be checked for accuracy against auscultatory BP measured by a general practitioner (GP) or other health professional, either ‘sometimes’ (*n* = 113, 54%) or ‘always’ (*n* = 31, 15%). Most respondents ensured people were supplied with an appropriately sized cuff ‘always’ (*n* = 140, 67%) or ‘sometimes’ (*n* = 54, 26%).

### Measuring BP in community pharmacy

Most respondents reported their pharmacy offers a service to measure BP (*n* = 178, 86%). If a BP measurement service was offered, one brand of BP device was reported to be used the most (*n* = 144/165 responses, 87%) and there was no evidence that BP kiosks were used in the pharmacies. Respondents ‘always’ provided the BP results to patients (*n* = 177/177 responses, 100%), but did not frequently pass them onto the patients’ GPs (*n* = 62, 36%). Many respondents also recorded the BP measurements in the pharmacy’s clinical information system (*n* = 112, 64%).

Respondents nearly always gave advice or recommendations to people following BP measurement (*n* = 176, 99%), which was mainly to take the results to a GP for interpretation (*n* = 134, 64%). Advice was also frequently given to people based on Australian BP guidelines [19] (*n* = 139, 66%).

## Discussion

This study found that pharmacists have a strong level of care about providing accurate BP devices to their consumers, but many were unaware that pharmacies stock mostly non-validated BP devices. There was low awareness of the factors associated with BP device accuracy and methods to determine if a device was accurate. While these findings are concerning, they are not unexpected because it is reasonable for pharmacists to expect that BP devices approved for sale by regulatory authorities have been appropriately tested for accuracy.

Automated BP devices need to be clinically validated to ensure that measurements are accurate [9, 10]. There are significant health ramifications of inaccurate BP measurement for individuals, as well as at the population-level. For individuals, underestimation of true BP may deprive an opportunity to reduce cardiovascular risk. On the other hand, overestimation of true BP could trigger unnecessary labelling of hypertension and prescription of lifelong antihypertensive treatment. For population-level health outcomes, one study using a large dataset from a BP survey conducted in China showed inaccurate BP measurements of just 6/3 mmHg could alter the perceived prevalence of hypertension from 33.4 to 44.7% and also change the perceived hypertension control rates from 9.7 to 6.0% [20]. These problems may influence individual health and public health policies on hypertension, but can be mitigated by using clinically validated BP devices and employing standardised BP measurement protocols [21].

Community pharmacies in Australia play a crucial role in patient health, including for the sale and provision of advice on BP devices to the general public. This survey has identified two important areas that require ongoing work. First, increased awareness and education of pharmacists about BP devices, which has also been identified in other countries [22–24]. One survey of 109 pharmacies from the United Kingdom identified substantial variability in the training of pharmacists to carry out BP monitoring services [24]. That study also identified that only one cuff size was available for 46% of pharmacy BP monitoring services. This finding may indicate knowledge deficits regarding the importance of cuff size to accurate BP measurement, but it is unknown whether the devices that were sold by the pharmacies also had limited cuff sizes available. Consistent with the present study, previous work has also found limited awareness or consideration of validation status of BP devices available or used in pharmacies in Turkey and the United Kingdom [22, 24]. Pharmacist education could be facilitated via professional organisations and through accreditation pathways and there is evidence of successful educational interventions related to understanding reference values for BP [25]. Education should help rectify apparent misconceptions held by some pharmacists about BP devices, such as particular brands being more accurate than others, as identified in Fig. 3.

The second important area for ongoing work is dedicated advocacy for policy changes, including regulations and strategic action, to influence supply chains to ensure only validated BP devices are sold in Australian pharmacies. Influencing supply chains could be facilitated via pharmacy owners and chains, because these stakeholders strongly influence which BP devices are stocked by pharmacies. In the present study, 36% of respondents identified pharmacy chains as responsible for decisions about what BP devices are stocked. This is similar to a previous English study which found 43% of BP devices used in pharmacy BP monitoring services were provided by the head office of the business [24]. Fifty percent of respondents also selected “Quality” as a reason for devices to be stocked, and this finding is potentially driven by other factors such as brand recognition and marketing of certain devices by manufacturers. The World Health Organisation has also recommended that government regulations should be strengthened to require that all BP devices are validated for accuracy prior to pre-market approval [5, 26]. Increased awareness of pharmacists and consumers on the importance of clinical validation should also influence manufacturers as sales become preferentially favoured towards validated BP devices. As well as validation of BP devices, the BP measurement protocols that pharmacists use to measure BP are an important component of accurate BP measurement but questions on this topic were beyond the scope of this survey.

Beyond validation issues related to BP devices, pharmacists should also provide standardised advice to consumers at the point of sale on how to use their BP device and measure BP properly at home. Ideally, pharmacists should ensure that consumers are supplied with an appropriately sized cuff for their arm and, reassuringly, most respondents said they do this either ‘all’ or ‘some of the time’. Most respondents also provided advice to consumers on how to measure BP at home. However, variable sources of evidence were drawn on to provide this advice, with 70% of respondents stating this was based on their general knowledge on BP.

Deviations from internationally accepted home BP protocols could lead to systematic errors in measurements. Therefore, these findings suggest more work can be done to standardise the sources of advice for consumers and ensure that only rigorous, evidence-based materials are used. Best practice resources currently exist from the Australian home BP consensus statement [19], including pragmatic materials [27], which were endorsed by the Heart Foundation and High BP Research Council of Australia, but may need to be tailored specifically for pharmacists to effectively deliver to their consumers.

There are some study limitations. The study sample size was relatively small and we cannot exclude the possibility of selection bias or that the respondents were not representative of Australian pharmacists. The precise response rate could not be calculated because the number of people receiving the invitation via the PSA email distribution could not be provided. Although the number of study participants may be modest, the characteristics of the respondents appeared to be demographically representative of Australian pharmacists, according to available national statistics, in terms of age, sex and location [18]. Furthermore, selection bias could be positive, meaning that pharmacists with an interest in BP responded. If this was the case, the results may underestimate the problems related to pharmacist awareness of issues related to BP device validation and accuracy. The survey was not designed to determine the specific advice that pharmacists provide to consumers; therefore, it is uncertain the extent to which this advice is appropriate. Nevertheless, diverse sources of advice were identified, which suggests there is a need for standardisation. Whether the findings from this study are generalisable globally, especially to countries with different health systems or pharmacy training to Australia, or to lower-income countries remains uncertain. Studies from investigators outside of Australia indicate that community pharmacies are an important source of BP devices and BP monitoring for the public [28–30].

## Conclusions

Australian pharmacists have a high level of care with respect to providing accurate BP devices to consumers, but they were generally unaware, and justifiably surprised, that most devices available from pharmacies were not validated for accuracy according to rigorous international standards. This survey demonstrates the need for education, dedicated advocacy for policies, including regulations and strategic action, to ensure only validated BP devices are sold in Australian pharmacies and that evidence-based advice related to BP measurement is provided to consumers.

## Summary table

### What is known about this topic

- A recent study found less than a quarter of BP devices available from Australian pharmacies were validated for accuracy
- Pharmacists are trusted health professionals in Australia that supply BP devices to Australian consumers, but whether they are aware of the low validation rate is unknown.

### What this study adds

- Via online survey, we found that the accuracy of devices sold by Australian pharmacists was quite or extremely important to most respondents.
- Most respondents were unaware of only 23.8% of devices sold by Australian pharmacists were validated and there was low awareness on how to identify accurate devices and other factors related to device accuracy.
- The findings indicate there is a need for education and advocacy toward exclusive sale of validated BP devices in Australian pharmacies.

## Supplementary information

Supplemental material

Supplemental Results

## Data Availability

The datasets analysed during the current study are available from the corresponding author on reasonable request.
