# Supplementary material for: Perceptions of pharmacists on the quality of automated blood pressure devices: a national survey
Source: J Hum Hypertens. 2022 Mar 21;37(3):235–40. doi: 10.1038/s41371-022-00670-4 (PMC9995266; doi:10.1038/s41371-022-00670-4)
Supplement: Supplementary file 1 — Supplemental material [file 41371_2022_670_MOESM1_ESM.pdf]

Survey of pharmacists on the quality of blood pressure devices provided in Australian pharmacies

Australian guidelines recommend that people purchase a blood pressure device for home monitoring, and a common place of purchase is pharmacies. Little is known on the quality of blood pressure devices supplied through Australian pharmacies and this survey seeks to better understand this from the views of practicing pharmacists.

By filling out the survey, which takes about 10 minutes, you are providing consent for your responses to be used for research.

You can also go into a draw to win a $500 gift voucher.The survey is a study carried out by researchers from the University of Tasmania, which has been approved by the Human Research Ethics Committee of Tasmania (H0023506).

You can find out more study information here: https://cloudstor.aarnet.edu.au/plus/s/jvdsK5BUXMNNbYQ

The first few questions are to ascertain non-identifiable demographic information.

Age range	◯ Less than 30 years<br>◯ 30-39 years<br>◯ 40-49 years<br>◯ 50-59 years<br>◯ 60-69 years<br>◯ 70 years or over<br>◯ Prefer not to say
Sex	◯ Female<br>◯ Male<br>◯ Prefer not to say
Highest pharmacy qualification	◯ B.Pharm<br>◯ Honours (Hons)<br>◯ PhD<br>◯ Masters e.g. M Pharm, M Clin Pharm<br>◯ Ph.C<br>◯ Grad.Dip<br>◯ Other ______
The period in which you initially registered was:	◯ Prior to 1985<br>◯ 1986 to 2000<br>◯ 2001-2015<br>◯ 2016 or later
The country you initially registered in?	◯ Australia<br>◯ Overseas
Are you accredited to conduct HMRs/RMMRs?	◯ No<br>◯ Yes<br>◯ Currently enrolled
Which state/territory of Australia do you primarily practice in?	◯ ACT<br>◯ QLD<br>◯ TAS<br>◯ VIC<br>◯ NSW<br>◯ SA<br>◯ WA<br>◯ NT

Practice details

In which area of practice do you spend the majority of your time in most weeks?	◯ Community Pharmacy<br>◯ Hospital Pharmacy<br>◯ Teaching or research<br>◯ Consultant<br>◯ Other ______
How would you describe your main position?	◯ Pharmacy Owner<br>◯ Pharmacy Manager<br>◯ Employee pharmacist<br>◯ Locum<br>◯ Consultant<br>◯ Retired<br>◯ Teaching or research<br>◯ Working, but not within pharmacy<br>◯ Other ______

Details of place of work
If you are not currently involved in community pharmacy in some capacity please skip this section and go to the blood pressure device questions on the next page.
This information helps us to understand survey responses from people of similar experience and working environments. Please provide the following information for your primary place of employment.

Postcode of the pharmacy you work in: ______________________________

Number of pharmacists usually working within the practice:
- ◯ 1-2
- ◯ 3-4
- ◯ 5+

Number of hours open each week:
- ◯ < 50
- ◯ 50 - 70
- ◯ > 71

Average number of prescriptions dispensed each week:
- ◯ ≤ 500
- ◯ 501 - 1200
- ◯ 1201 - 3000
- ◯ > 3000
- ◯ Unsure

Blood pressure device questions

Is the accuracy of blood pressure devices sold by pharmacists an issue of importance to you?	◯ Not at all important<br>◯ A little important<br>◯ Neutral<br>◯ Quite important<br>◯ Extremely important
Do you consider all blood pressure devices sold in your pharmacy to be equally reliable in terms of measurement accuracy?	◯ No<br>◯ Yes<br>◯ Unsure
True or false, before being sold in Australia, blood pressure devices must pass accuracy testing according to rigorous scientific standards:	◯ True<br>◯ False<br>◯ Unsure<br>◯ Other ______
True or false, if a blood pressure device has been approved for sale by the Therapeutic Goods Administration, this is confirmation that it has passed accuracy testing according to rigorous scientific standards:	◯ True<br>◯ False<br>◯ Unsure<br>◯ Other ______
What factors could be used to identify the accuracy of a blood pressure device? (check all that apply)	☐ Brand<br>☐ Type (e.g. upper-arm device vs. wrist device)<br>☐ Price (e.g. more expensive devices are more likely to be accurate)<br>☐ Claims of clinical accuracy on the box or via other marketing<br>☐ Checks of online databases that list accurate blood pressure devices<br>☐ Unsure<br>☐ None of the above<br>☐ Other ______
Which type of blood pressure device is usually more accurate?	◯ Upper-arm<br>◯ Wrist<br>◯ Both upper-arm and wrist devices are equally accurate<br>◯ Unsure
Do you associate a particular brand of blood pressure device with being more accurate than others?	◯ No<br>◯ Yes<br>◯ Unsure
Which of the following brands of blood pressure devices do you associate with being more accurate than others? (check all that apply)	☐ A&D Medical<br>☐ Beurer<br>☐ Dr. Trust<br>☐ iHealth<br>☐ Lot Fancy<br>☐ Microlife<br>☐ Omron<br>☐ Welch Allyn<br>☐ Other ______
When people buy a blood pressure device at your pharmacy, are they provided with any advice or information on how to measure blood pressure at home?	◯ No<br>◯ Yes, always<br>◯ Yes, sometimes

What sources of evidence are used for the advice or information on how to measure blood pressure at home? (check all that apply)

- ☐ General knowledge on home blood pressure measurement
- ☐ Australian home blood pressure measurement expert consensus statement
- ☐ National Heart Foundation of Australia information
- ☐ European Society of Hypertension guidelines
- ☐ American Heart Association information
- ☐ Unsure
- ☐ Other ______

Do you recommend people take their blood pressure device to be checked for accuracy against manual measurements by a GP or other health professional?

- ◯ No
- ◯ Yes, always
- ◯ Yes, sometimes

With each blood pressure device that is purchased, do you ensure that an appropriately sized blood pressure cuff is supplied?

- ◯ No
- ◯ Yes, always
- ◯ Yes, sometimes

Do you offer a service to measure blood pressure at your pharmacy?

- ◯ No
- ◯ Yes
- ◯ Unsure

What device brand do you use for measuring blood pressure at your pharmacy? (if unsure please leave blank)

____

What device model do you use for measuring blood pressure at your pharmacy? (if unsure please leave blank)

____

Are the blood pressure measures taken at your pharmacy:	No	Yes	Unsure
Provided to the person	◯	◯	◯
Provided to a GP or other health professional	◯	◯	◯
Recorded in a pharmacy database	◯	◯	◯

After measuring blood pressure at your pharmacy, do you provide advice or recommendations to people?

- ◯ No
- ◯ Yes
- ◯ Unsure

What advice or recommendations are given to people? (check all that apply)

- ☐ Advised to take their results to their GP for interpretation
- ☐ Advice or recommendations based on Australian blood pressure guidelines
- ☐ Other ______

On what basis do you select which blood pressure devices to stock? (check all that apply)	☐ To ensure a range of different prices<br>☐ To ensure there are different types (upper-arm and wrist)<br>☐ Quality<br>☐ Brands<br>☐ Accuracy<br>☐ Additional functions (e.g. Bluetooth, storage of measurements)<br>☐ Determined by pharmacy banner group<br>☐ None of the above<br>☐ Other ______
Were you aware that only 23.8% of blood pressure devices sold by pharmacies in Australia have passed accuracy testing according to rigorous scientific standards?	◯ No<br>◯ Yes<br>◯ Unsure
Is this statistic surprising to you?	◯ Not at all surprising<br>◯ A little surprising<br>◯ Neutral<br>◯ Quite surprising<br>◯ Extremely surprising

Thank you for completing this survey.
After clicking submit you will be automatically directed to a final page and if you would like to enter the draw to win a $500 gift voucher please enter your email address (this will only be used to contact you if you are the prize winner).
