# Supplementary material for: Perceptions of pharmacists on the quality of automated blood pressure devices: a national survey
Source: J Hum Hypertens. 2022 Mar 21;37(3):235–40. doi: 10.1038/s41371-022-00670-4 (PMC9995266; doi:10.1038/s41371-022-00670-4)
Supplement: Supplementary file 2 — Supplemental Results [file 41371_2022_670_MOESM2_ESM.docx]

| Supplementary Table 1. Additional characteristics of the 210 pharmacists who completed the survey. | |
| --- | --- |
| Characteristics | n (%) |
| Country initially registered, Australia (vs. overseas) | 180 (86) |
| Accredited to conduct HMRs/RMMRs |  |
| No | 156 (75) |
| Yes | 47 (22) |
| Currently enrolled | 6 (3) |
| Area of practice the majority time spent most weeks |  |
| Community pharmacy | 181 (86) |
| Hospital pharmacy | 12 (6) |
| Teaching or research | 3 (1) |
| Consultant | 8 (4) |
| Other | 6 (3) |
| Main position |  |
| Pharmacy owner | 33 (16) |
| Pharmacy manager | 42 (20) |
| Employee pharmacist | 94 (45) |
| Locum | 17 (8) |
| Consultant | 10 (5) |
| Retired | 1 (0) |
| Teaching or research | 3 (1) |
| Working, but not within pharmacy | 3 (1) |
| Other | 7 (3) |
| *Community pharmacy characteristics* |  |
| Number of pharmacists working in the practice |  |
| 1-2 | 106 (53) |
| 3-4 | 62 (31) |
| 5+ | 31 (16) |
| Pharmacy opening hours per week |  |
| <50 | 51 (26) |
| 50 to 70 | 92 (46) |
| Over 70 | 56 (28) |
| Prescriptions dispensed per week |  |
| ≤ 500 | 38 (19) |
| 501 to 1200 | 59 (30) |
| 1201 to 3000 | 69 (35) |
| >3000 | 14 (7) |
| Unsure | 19 (10) |
